# Caudate nucleus volume in medicated and unmedicated patients with early- and adult-onset schizophrenia

**DOI:** 10.1038/s41598-024-73322-x

**Published:** 2024-10-01

**Authors:** Dimitrios Andreou, Kjetil Nordbø Jørgensen, Stener Nerland, Tereza Calkova, Lynn Mørch-Johnsen, Runar Elle Smelror, Laura A. Wortinger, Mathias Lundberg, Hannes Bohman, Anne Margrethe Myhre, Erik G. Jönsson, Ole A. Andreassen, Ingrid Agartz

**Affiliations:** 1 Department of Psychiatric Research, Diakonhjemmet Hospital, Forskningsveien 7, 0373 Oslo, Norway; 2 Norwegian Centre for Mental Disorders Research (NORMENT), Institute of Clinical Medicine, University of Oslo, Oslo, Norway; 3 Centre for Psychiatry Research, Department of Clinical Neuroscience, Karolinska Institutet & Stockholm Health Care Services, Stockholm Region, Stockholm, Sweden; 4 Division of Mental Health and Addiction, Vestre Viken Hospital Trust, Drammen, Norway; 5 Centre for Clinical Research, Vastmanland Hospital Vasteras, Region Vastmanland – Uppsala University, Västerås, Sweden; 6 Department of Psychiatry, Department of Clinical Research, Østfold Hospital, Grålum, Norway; 7 Department of Clinical Science and Education, Södersjukhuset, Karolinska Institutet, Stockholm, Sweden; 8 Department of Neuroscience, Child and Adolescent Psychiatry and Psychiatry Unit, Uppsala University, Uppsala, Sweden; 9 Division of Mental Health and Addiction, Departement of Research and innovation, Oslo University Hospital, Oslo, Norway; 10 Child and Adolescent Psychiatry Unit, Institute of Clinical Medicine, University of Oslo, Oslo, Norway; 11 Division of Mental Health and Addiction, Norwegian Centre for Mental Disorders Research (NORMENT), Oslo University Hospital, Oslo, Norway

**Keywords:** Caudate, MRI, Schizophrenia, Early-onset, Adult-onset, Antipsychotics, Schizophrenia, Brain

## Abstract

The caudate nucleus is a part of the striatum, and striatal hyperdopaminergia is considered central to the pathophysiology of schizophrenia. How caudate volume is affected in schizophrenia and what role antipsychotics play remains unclear. In early-onset schizophrenia (EOS), where psychosis emerges during a neurodevelopmentally critical phase, the caudate may exhibit a heightened vulnerability to the effects of antipsychotic medications. We hypothesized effects of both antipsychotic medication use and age of onset on caudate in schizophrenia. We included adult patients with EOS (*n* = 83) and adult-onset schizophrenia (AOS) (*n* = 246), adult healthy controls (HC, *n* = 774), adolescent patients with non-affective psychosis (*n* = 56) and adolescent HC (*n* = 97). We obtained T1-weighted MRI scans using a 1.5T Siemens scanner and General Electric 3T scanners. In our main analysis, we tested for main and interaction effects of diagnosis and current antipsychotic medication use on caudate volume. Adult patients with EOS (*p* < 0.001) and AOS (*p* = 0.002) had both larger caudate than HC. Age of onset (EOS/AOS) interacted with antipsychotic use (*p* = 0.004) which was associated with larger caudate in EOS (*p* < 0.001) but not in AOS (*p* = 0.654). Conversely, among medicated patients only, EOS had larger caudate than AOS (*p* < 0.001). No other subcortical structures showed differences between medicated EOS and AOS. Medicated adolescent patients with non-affective psychosis and medicated adult patients with EOS showed similar caudate volumes. The results may indicate a schizophrenia-related and a medication-induced caudate increase, the latter restricted to patients with EOS and possibly occurring already in adolescence shortly after disease onset.

**Supplementary Information:**

The online version contains supplementary material available at 10.1038/s41598-024-73322-x.

## Introduction

Schizophrenia (SZ) is a chronic brain disorder considered to result from a complex interplay between genetic and environmental risk factors, and constitutes a significant contributor to the global health burden and functional impairment^1,2^. SZ typically manifests during early adulthood. Nevertheless, a subset of patients (5–18%) experiences the first psychotic episode during childhood or adolescence; the disorder is then termed early-onset SZ (EOS), and research data suggest that it tends to be associated with a less favorable prognosis in comparison to the adult-onset SZ (AOS)^3,4^.

The dopamine hypothesis stands as one of the most foremost theories in understanding SZ. While it’s important to note that SZ involves other neurotransmitters such as glutamate and serotonin, a substantial body of evidence underscores the pivotal role of dopamine in both the pathophysiology and treatment of the disorder^5–7^. Within this framework, positive symptoms in SZ are commonly attributed to striatal hyperdopaminergia, whereas negative symptoms may be linked to frontal hypodopaminergia^7,8^. The striatum can be divided into two main regions: a dorsal region consisting of the caudate nucleus and the putamen, and a ventral region comprising the nucleus accumbens and the olfactory tubercle^9^. The dorsal striatum plays a critical role in regulating motor functions and cognitive processes; specifically, the caudate nucleus appears to regulate higher goal-directed actions, while the putamen is involved in simpler behaviors^10,11^. The ventral striatum is implicated in reward processing and motivation^9^.

Caudate nucleus has been extensively studied in SZ, but with conflicting results; volumetric studies have shown both similar, smaller and larger caudate volumes in patients compared to HC^12–15^. A body of evidence implicates antipsychotic use in caudate volume alterations in SZ, with some, but not all, studies showing a medication-related caudate enlargement^12,13,16^. Importantly, there is evidence from animal studies showing antipsychotic-induced striatal enlargement^17,18^, an effect that partly reverses after medication discontinuation^18^. Further, caudate decreases linearly with age during adulthood^19^, while during childhood and adolescence, although the literature is rather conflicting, an inverted U-shaped trajectory has been reported^20^. The dynamic environment during adolescence, the second period of major neurodevelopment where the brain is still maturing^21^, may render the brain in general and the caudate in particular susceptible to environmental factors such as antipsychotic medication use. Specifically, the dopamine system undergoes substantial developmental changes during adolescence^22,23^ and these include changes in striatal dopamine receptor density and function^24,25^. Although the functional significance of these changes remains to be fully clarified, striatal plasticity during adolescence is thought to support learning and decision-making during a critical life phase^26^. Preclinical studies comparing effects of antipsychotic treatment on locomotor activity have shown differential responses in adolescence compared to adulthood^27,28^, where a compensatory hyperactivity after treatment cessation persisted in adolescent rats but not in adult rats^28^. It is plausible that volumetric changes within the striatum following antipsychotic treatment might also differ in adolescence compared to adulthood, but this hypothesis has to our knowledge not been tested directly.

Here, we used magnetic resonance imaging (MRI) to assess caudate volumes in adult patients with EOS, AOS and healthy controls (HC), and in adolescents with non-affective psychotic disorders and adolescent HC. We hypothesized that both antipsychotic use and the age of onset would be associated with caudate volume. More specifically, we anticipated a medication-related increase in caudate volume among early-onset patients, while expecting no, or a lesser, increase, among adult-onset patients. To determine if the putative effects were specific to the caudate, we also examined the volumes of other subcortical grey matter structures, including the putamen, nucleus accumbens, pallidum, thalamus, amygdala and hippocampus.

## Subjects and methods

### Participants

We included an adult Norwegian sample, an adolescent Norwegian sample and an adolescent Swedish sample in this study. For the Norwegian samples, patients were recruited from outpatient and inpatient psychiatric units in the Oslo region, Norway. HC were recruited from the same catchment area using the Norwegian population registry. These participants were recruited as part of the Thematically Organized Psychosis (TOP) study and the Youth-TOP study conducted within the Norwegian Centre for Mental Disorders Research (NORMENT, Oslo, Norway; www.med.uio.no/norment/english). Medical doctors and psychologists assessed the adult patients with the Structured Clinical Interview (SCID-I) for the Diagnostic and Statistical Manual of Mental Disorders, fourth edition (DSM-IV)^29^. HC were screened for mental disorders with the Primary Care Evaluation of Mental Disorders (Prime-MD)^30^. Medical doctors and psychologists assessed the adolescent patients and HC with the Schedule for Affective Disorders and Schizophrenia for School Aged Children – Present and Lifetime Version (K-SADS-PL)^31^. For the Swedish adolescent sample (Stockholm Child and Adolescence Psychosis Study (SCAPS), Karolinska Institutet, Stockholm, Sweden), patients were recruited from the psychosis and bipolar disorder unit, Child and Adolescent Psychiatry Clinic, Stockholm, Sweden. Patients were assessed by child- and adolescent psychiatry specialists working in the clinic. HC were recruited from the same catchment area using the Swedish population registry. Research diagnoses, according to DSM-IV, were established in agreement by two clinical experts (EGJ; DA) based on the patients’ medical records.

We included 329 adult patients with SZ spectrum disorders, i.e., SZ (*n* = 240), schizophreniform disorder (*n* = 31) or schizoaffective disorder (*n* = 58), and 774 adult HC, aged 18–65 years, as well as 56 adolescent patients (Norwegian sample: 37 patients; Swedish sample: 19 patients) with early-onset non-affective psychosis (onset of illness < 18 years of age)^32^, i.e., SZ (*n* = 21), schizoaffective disorder (*n* = 1), brief psychotic disorder (*n* = 1) or psychotic disorder not otherwise specified (*n* = 33), and 97 adolescent HC (Norwegian sample: 70 HC; Swedish sample: 27 HC), aged 12–18 years. The age of onset distribution for adult and adolescent patients as well as the age distribution for adult and adolescent patients and HC are shown in Suppl. Figure 1 & Suppl. Figure 2.

Exclusion criteria for all participants: previous moderate or severe head injury, neurological disorders or medical conditions thought to affect brain function. HC with previous or current psychiatric disorders including substance use disorder (including alcohol use disorder) or with first-degree relatives with severe mental disorders were excluded. Adolescent patients who met the criteria for substance use disorder (including alcohol use disorder) were also excluded. In this context, adolescent patients with regular use of illicit drugs (including cannabis) or alcohol were excluded; however, those with sporadic use of illicit drugs or alcohol were included.

The study was approved by the Regional Committee for Medical Research Ethics South East Norway (REC South East) and the Norwegian Data Inspectorate, and was conducted in accordance with the Declaration of Helsinki as revised in 2008. We obtained written informed consent from all participants. For participants below the age of 16 informed consent was obtained from the parents of all the participants.

Data supporting the findings of the present study have repository at NORMENT/Oslo University Hospital. Restrictions apply to the availability of data and are thereby not publicly available. Data can be made available under reasonable request and with permission of NORMENT/Oslo University Hospital, in accordance with the ethics agreements/research participants consent.

### Clinical measures

We assessed the adult and the Norwegian adolescent patients with the Positive and Negative Syndrome Scale (PANSS)^33^. We assessed the current use of antipsychotic medications by interviews and review of medical records, and calculated the current chlorpromazine equivalent doses (CPZ) in mg/day^34^. We defined the age of onset as the age at first psychotic episode. For adults, we evaluated alcohol use with the Alcohol Use Disorder Identification Test (AUDIT)^35^ and drug use with the Drug Use Disorder Identification Test (DUDIT)^36^.

#### Magnetic resonance imaging

T1-weighted MRI scans were acquired on a 1.5T Siemens MAGNETOM Sonata scanner (Siemens Medical Solutions, Erlangen, Germany) with a standard head coil (205 adult patients and 291 adult HC), a 3T General Electric Signa HDxt scanner (GE Medical Systems, Milwaukee, USA) with an 8-channel head coil (73 adult patients, 278 adult HC, 19 adolescent patients and 36 adolescent HC), and two General Electric 3T Discovery MR750 scanners with 32-channel head coils (51 adult patients, 205 adult HC, 37 adolescent patients and 61 adolescent HC). For the 1.5T Siemens MAGNETOM Sonata, two T1-weighted scans were obtained and averaged to increase signal-to-noise ratio (SNR). See Suppl. Table 8 for an overview of the scanner systems used to acquire each sample and their T1-weighted MRI sequences.

MRI scans were processed using FreeSurfer v6.0.0 ^37^. In the absence of a specific hypothesis regarding laterality, we utilized the combined volumes (sum) of the left and right caudate for all analyses (this approach yields equivalent results to using the average of the two volumes). However, for one post-hoc analysis titled “Caudate specificity and laterality in EOS vs. AOS medicated adult patients,” we analyzed the left and right volumes separately for all examined subcortical structures, including the caudate, putamen, nucleus accumbens, pallidum, thalamus, amygdala, and hippocampus volumes (https://surfer.nmr.mgh.harvard.edu/fswiki/MorphometryStats). All T1-weighted (T1w) images were inspected by trained research assistants and excluded if major artifacts, e.g., due to excessive movement, were present. Surface reconstructions were manually edited in the event of reconstruction errors following standard FreeSurfer procedures. Voxel-wise segmentations (ASEG) were routinely inspected to rule out segmentation errors. Although manual editing was not performed for the ASEG segmentation, it is important to note that the caudate has excellent contrast in T1w images and that the ASEG segmentation of the caudate has shown excellent within- and between-version reliability^38^ and high overlap with manual segmentations^39^. A visualization of caudate segmentations is shown in Suppl. Figure 4.

### Statistics

#### Main analysis

Adult patient analysis: We assessed group differences between adult patients with EOS and AOS in sex, age, duration of illness (DOI), duration of untreated psychosis (DUP), AUDIT and DUDIT scores, PANSS total score, current antipsychotic medication use and CPZ as well as their correlations with caudate volume separately for EOS and AOS (Table 1).

**Table 1:** Group differences between adult patients with early-onset schizophrenia (EOS) and patients with adult-onset schizophrenia (AOS) in sex, age, duration of illness (DOI), duration of untreated psychosis (DUP), alcohol use disorder identification test (AUDIT) score, drug use disorder identification test (DUDIT) score, positive and negative syndrome scale (PANSS) total score, the percentage of patients on antipsychotics as well as the chlorpromazine equivalent doses (CPZ) among patients on antipsychotics.

	EOS		AOS		*P*-value^b^	EOS: Correlations with caudate volume		AOS: Correlations with caudate volume	
	*N* ^a^	Mean (SD) or %	*N* ^a^	Mean (SD) or %		+/-	*P*-value^c^	+/-	*P*-value^c^
Sex (% women)	83	47	246	38.6	0.180	-^4^	**0.003**	-^d^	**< 0.001**
Age (years)	83	26.1 (8.1)	246	32.8 (9.1)	**< 0.001**	-	0.857	-	0.425
DOI (years)	83	10.5 (8.3)	246	7.1 (6.8)	**< 0.001**	-	0.199	-	0.698
DUP (days)	67	216.6 (293.3)	165	110.1 (234.5)	**0.009** ^**e**^	-	0.759	+	0.461
AUDIT	67	7.3 (7.5)	181	6.1 (5.9)	0.196	+	0.757	+	0.258
DUDIT	68	4.3 (8.4)	190	3.9 (7)	0.746	+	0.976	+	0.246
PANSS total score	81	62.1 (15)	243	60.4 (17.1)	0.448	-	0.338	+	0.428
On antipsychotics (%)	83	89.2	246	89.4	0.994	+	**0.003**	+	0.564
CPZ (mg/day)	74	346.5 (231.6)	217	367.6 (283.1)	0.563	+	**0.033**	-	0.507

Correlations of each variable with the caudate nucleus volume for EOP and AOP patients separately are also shown. P values < 0.05 shown in bold.

^a^Number of participants with data for each variable.

^b^Chi-square test or t-test.

^c^Point-biserial correlations for binary variables; Spearman’s correlations for quantitative variables.

^d^Women had smaller caudate volume than men.

^e^Mann-Whitney U test was also run due to unequal variances, and confirmed the t-test result (*p* < 0.001).

Applying analyses of covariance (ANCOVAs) adjusted for sex, age, scanner and estimated total intracranial volume (ICV), we first explored the main effect of diagnostic status (EOS/AOS) on caudate (model 1), and we then explored the main and interaction effects of diagnostic status and antipsychotic medication use on caudate (model 2; main model). Due to the significant interaction effect (as thoroughly described in the “Results” section, there was a significant EOS/AOS-by-antipsychotic medication interaction (*p* = 0.004) on caudate), we interpreted the simple main effect of antipsychotic medication use in patients with EOS and patients with AOS, and the simple main effect of diagnostic status in medicated and non-medicated patients. Therefore, we accepted statistical significance for the simple main effects at a Bonferroni-adjusted alpha level of 0.0125 (0.05/4) (Table 2). Further, we added into the model covariates that significantly differed between the two patient groups based on the bivariate analysis as shown in Table 1 (model 3). Next, we reanalyzed models 1 and 2, using the age of onset of psychosis as a continuous variable instead of the binary EOS/AOS variable. Finally, we reanalyzed model 2, first with the EOS/AOS variable and then with the continuous age of onset of psychosis variable, with multivariate analyses of covariance (MANCOVAs) on accumbens, amygdala, caudate, hippocampus, pallidum, putamen and thalamus.

**Table 2:** The results of the two-way analysis of covariance (ANCOVA) on caudate volume among patients with schizophrenia.

ANCOVA with simple main effect analysis on caudate volume	F	*P*-value	Partial eta^2^	Estimated mean caudate volumes (mm^3^)	
EOS/AOS	0.262	0.609	0.001		
Use of antipsychotics	11.110	**< 0.001**	0.034		
EOS/AOS-by-use of antipsychotics	8.360	**0.004**	0.025		
Age	0.006	0.937	0.000		
Sex	0.609	0.436	0.002		
Scanner	11.761	**< 0.001**	0.068		
ICV	108.363	**< 0.001**	0.253		

Simple main effect analysis					
				**AP+**	**AP-**
AP+/AP- on caudate among patients with EOS	13.019	**< 0.001**	0.039	7939	6972
AP+/AP- on caudate among patients with AOS	0.202	0.654	0.001	7572	7502

				**EOS**	**AOS**
EOS/AOS on caudate among AP + patients	11.882	**< 0.001**	0.036	7939	7573
EOS/AOS on caudate among AP- patients	3.185	0.075	0.010	6972	7502

There was a significant EOS/AOS-by-antipsychotic use interaction on caudate (*p* = 0.004) which we followed up with simple main effect analysis. Significant associations (*p* < 0.05) are shown in bold. For the simple main effects, statistical significance was accepted at the Bonferroni-adjusted alpha level of 0.0125. EOS: early-onset schizophrenia, AOS: adult-onset schizophrenia AP+: currently on antipsychotics, AP-: currently not on antipsychotics.

Adult and adolescent patient/control analysis: Demographics and clinical characteristics of the adult and the adolescent patients and HC are shown in Table 3. Applying sex-, age-, scanner- and ICV-adjusted ANCOVAs, we investigated the main effect of diagnostic status, i.e., SZ vs. HC, EOS vs. HC and AOS vs. HC for the adult sample, and non-affective psychosis vs. HC for the adolescent sample, on caudate.

**Table 3:** Demographics and clinical characteristics of adult patients with schizophrenia spectrum disorders and adult healthy controls as well as adolescent patients with non-affective psychosis and adolescent healthy controls.

	Patients		Healthy controls		
	*N* ^a^	Mean (SD) or %	*N* ^a^	Mean (SD) or %	*P*-value^b^
Adult sample					
Sex (% females)	329	40.7	774	44.3	0.271
Age (years)	329	31.1 (9.3)	774	34.3 (9.1)	**< 0.001**
ICV (cm^3^)	329	1557.1 (179.2)	774	1562.9 (162.9)	0.600
DOI (years)	329	7.9 (7.3)			
DUP (days)	232	140.8 (256.8)			
PANSS total score	324	60.8 (16.6)			
On antipsychotics (%)	329	89.4			
CPZ (mg/day)	291	362.2 (270.6)			
Adolescent sample					
Sex (% females)	56	62.5	97	61.9	0.937
Age (years)	56	16.3 (1.2)	97	16.2 (1.5)	0.743^c^
ICV (cm^3^)	56	1506.3 (178.2)	97	1541.5 (148.6)	0.192
DOI (years)	49	1.9 (1.8)			
DUP (days)	56	246.3 (317.6)			
PANSS total score^d^	36	73.2 (16.2)			
On antipsychotics (%)	51	70.6			
CPZ (mg/day)	51	227.9 (122.8)			

Group differences in sex distribution, age and estimated total intracranial volume (ICV) between patients and healthy controls are shown. For patients, the duration of illness (DOI), the duration of untreated psychosis (DUP), positive and negative syndrome scale (PANSS) total score, the percentage of patients on antipsychotics as well as the chlorpromazine equivalent doses (CPZ) among patients on antipsychotics are shown. P values < 0.05 shown in bold.

^a^Number of participants with data for each variable.

^b^Chi-square test or t-test.

^c^Mann-Whitney U test was also run due to unequal variances, and confirmed the t-test result (*p* = 0.943).

^d^Data only for the Norwegian sample.

#### Post-hoc analysis

Caudate specificity and laterality in EOS vs. AOS medicated adult patients: To determine caudate specificity and laterality, we ran post-hoc ANCOVAs of EOS/AOS status on left and right caudate, putamen, pallidum, thalamus, nucleus accumbens, hippocampus and amygdala volumes, whilst controlling for sex, age, ICV and scanner (Table 4). We applied a false discovery rate (FDR) of 5% by hemisphere to correct for multiple testing^40^.

**Table 4:** Estimated mean volumes and the corresponding p-values from the analyses of covariance (ANCOVAs) of early-onset schizophrenia/adult-onset schizophrenia (EOS/AOS) status on left and right subcortical volumes among adult patients with schizophrenia spectrum disorders currently on antipsychotics.

Volumes in mm^3^	Left hemisphere			Right hemisphere		
	EOS	AOS	*P*-value	EOS	AOS	*P*-value
N	74	220		74	220	
Accumbens	589	585	0.762	610	597	0.290
Amygdala	1584	1604	0.447	1780	1755	0.314
Caudate	3948	3756	< 0.001*	4001	3835	0.003*
Hippocampus	4091	4132	0.352	4181	4201	0.661
Pallidum	2151	2114	0.154	2146	2100	0.082
Putamen	5459	5378	0.218	5494	5401	0.141
Thalamus	7885	7854	0.704	7215	7229	0.852

Nominally significant associations (*p* < 0.05) are shown in bold.

*Survives false discovery rate (FDR) correction for multiple testing.

*p < 0.05.

Medicated and non-medicated adolescent patients vs. adolescent HC: We investigated putative differences in caudate between medicated adolescent patients and HC, and between non-medicated adolescent patients and HC, applying sex-, age-, scanner- and ICV-adjusted ANCOVAs.

Medicated adult patients with EOS vs. medicated adolescent patients: We investigated the putative difference in caudate volumes between medicated adult patients with EOS and medicated adolescent patients with non-affective psychosis, applying a sex-, age-, scanner- and ICV-adjusted ANCOVA.

We measured the effect sizes with partial eta-squared (η^2^)^41^. We performed the statistical analyses using IBM SPSS Statistics 28.

## Results

### Main analysis

#### Adult patient analysis

In the bivariate analysis, patients with EOS were on average seven years younger (*p* < 0.001), had three years longer DOI (*p* < 0.001) and 3 ½ months longer DUP (*p* = 0.009) compared to patients with AOS, assessed with t-tests (Table 1). Among both patients with EOS and AOS, women had smaller caudate than men (assessed with point-biserial correlations, r_pb_ = -0.319, *p* = 0.003 and r_pb_ = -0.326, *p* < 0.001 for EOS and AOS, respectively). In EOS only, medication use, r_pb_=0.322, *p* = 0.003, and CPZ, assessed with Spearman’s correlation, r_s_ = 0.248, *p* = 0.033, were both positively correlated with caudate volume (Table 1 & Suppl. Figure 3).

**Fig. 1:**
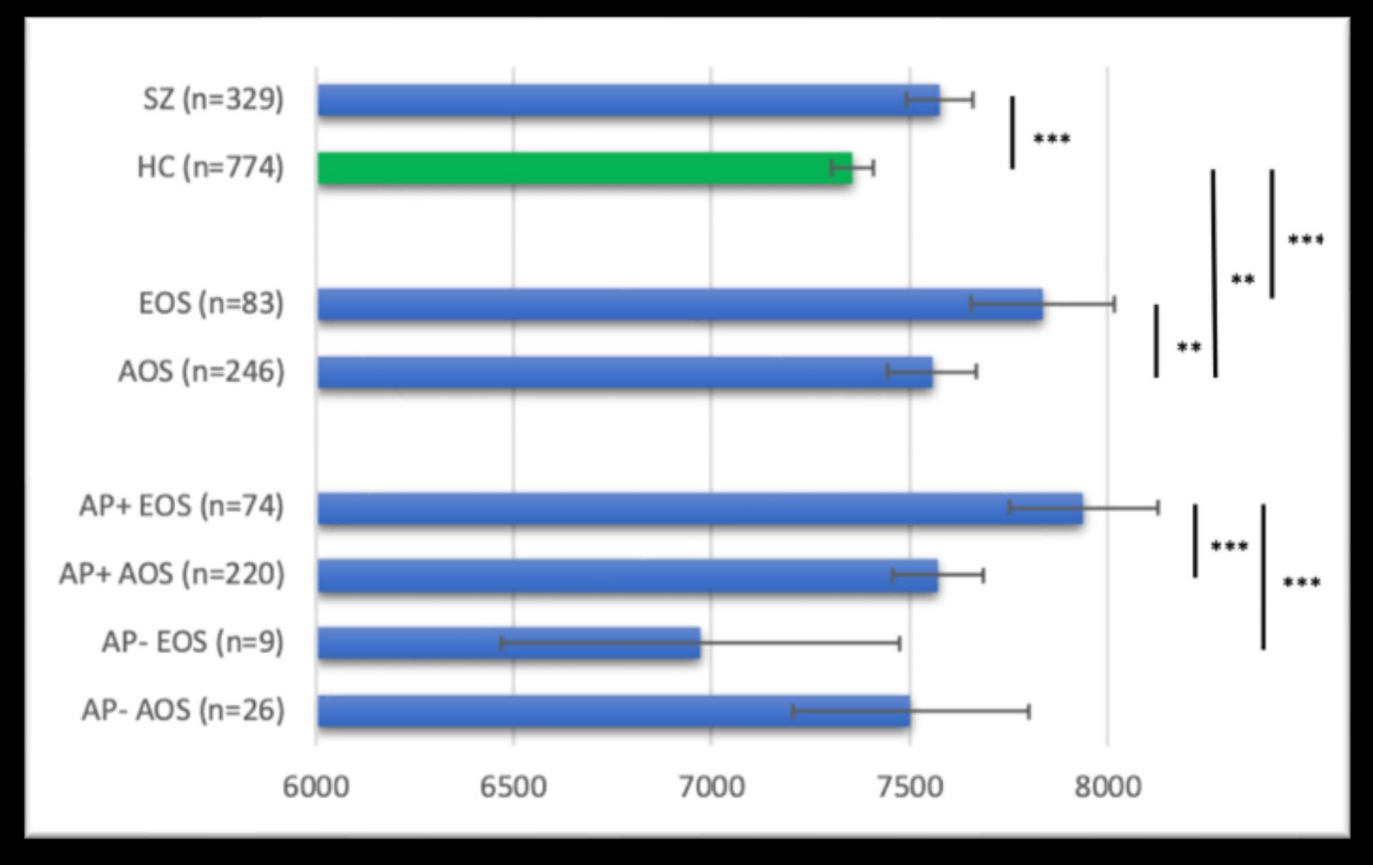
Caudate nucleus volume (in mm^3^) differences between adult patients with schizophrenia (SZ), early-onset SZ (EOS), adult-onset schizophrenia (AOS) and healthy controls (HC) (up), and between currently medicated with antipsychotics (AP+) and currently non-medicated with antipsychotics (AP-) adult patients with EOS and AOS (down). Adjusted means from age-, sex, scanner- and estimated total intracranial volume-adjusted analyses of covariance are shown. Error bars represent the 95% confidence intervals calculated using standard errors. The green bars represent the HC group, while the blue bars represent the patient groups. ***p < 0.001. **p < 0.01.

In the ANCOVA of diagnostic status (EOS/AOS) on caudate module (model 1; Fig. 1 & Suppl. Table 1), there was a statistically significant main effect of EOS/AOS, F(1,322) = 7.200, *p* = 0.008, η^2^ = 0.022. In the main ANCOVA (model 2; Fig. 1; Table 2), there was a significant EOS/AOS-by-antipsychotic medication interaction (*p* = 0.004) on caudate: among patients with EOS, there was a statistically significant effect of antipsychotic medication use on caudate, F(1,320) = 13.019, *p* < 0.001, η^2^ = 0.039, whereas among patients with AOS, there was no such effect, F(1,320) = 0.202, *p* = 0.654, η^2^ = 0.001. Specifically, adjusted mean caudate volumes in medicated patients with EOS were significantly larger than in non-medicated patients with EOS, a difference of 967 mm^3^ (95% CI, 440 to 1494), whereas adjusted mean caudate volumes in medicated and non-medicated patients with AOS did not differ (Fig. 1; Table 2). Conversely, among medicated patients, there was a statistically significant effect of EOS/AOS status on caudate volume, F(1,320) *=* 11.882, *p* < 0.001, η^2^ = 0.036, whereas among unmedicated patients, there was no such effect, F(1,320) = 3.185, *p* = 0.075, η^2^ = 0.010. Specifically, among medicated patients, adjusted mean caudate volume was significantly larger in EOS than in AOS, a difference of 366 mm^3^ (95% CI, 157 to 575), whereas among unmedicated patients, EOS and AOS patients did not significantly differ (Fig. 1; Table 2). Inserting DOI and DUP into the model (model 3; Suppl. Table 2), there was still a significant EOS/AOS-by-antipsychotic medication interaction (*p* = 0.008). Finally, EOS exhibited a significantly higher frequency of lifetime cannabis use compared to AOS (Suppl. Table 5). When the cannabis use variable was included in model 3, the interaction term remained significant (*p* = 0.012).

In the ANCOVA of the continuous age of onset of psychosis variable, age of onset was not associated with the caudate volume, F(1,322) = 0.647, *p* = 0.422, whilst controlling for sex, age, ICV and scanner (model 1). However, adding the antipsychotic medication variable and the age of onset-by-antipsychotic medication interaction term (model 2) we found a significant interaction (*p* = 0.004): among patients on antipsychotics, the age of onset was inversely (non-significantly) associated with caudate, F(1,287) = 3.061, *p* = 0.081, η^2^ = 0.011, whereas among patients not on antipsychotics, the age of onset was positively (non-significantly) associated with caudate, F(1,28) = 3.060, *p* = 0.091, η^2^ = 0.099.

We finally conducted two MANCOVAs on accumbens, amygdala, caudate, hippocampus, pallidum, putamen and thalamus. In the first MANCOVA we inserted age, sex, scanner, ICV, EOS/AOS, antipsychotic use and the interaction term EOS/AOS-by-antipsychotic use. In the second MANCOVA, we inserted age, sex, scanner, ICV, age of onset of psychosis (continuous), antipsychotic use and the interaction term age of onset of psychosis-by-antipsychotic use. In the first MANCOVA, there was no significant main effect of EOS/AOS (*p* = 0.948) or EOS/AOS-by-antipsychotic use interaction effect (*p* = 0.120). Similarly, in the second MANCOVA, there was no significant main effect of age of onset (*p* = 0.647) or age of onset-by-antipsychotic use interaction effect (*p* = 0.097). Follow-up analysis of the second MANCOVA showed that there was a significant age of onset-by-antipsychotic use interaction effect on the caudate (*p* = 0.004, also shown in the previous paragraph), but not on the accumbens (*p* = 0.086) amygdala (*p* = 0.584), hippocampus (*p* = 0.851), pallidum (*p* = 0.501), putamen (*p* = 0.078) or thalamus volumes (*p* = 0.401).

#### Adult patient/control analysis

In the bivariate analysis of the adult sample, patients (*n* = 329) were three years younger than HC (*n* = 774); patients and HC did not significantly differ in sex distribution or ICV (Table 3). In the sex-, age-, ICV-, and scanner-adjusted ANCOVA, patients had significantly larger caudate volume than HC, F(1,1096) = 19.372, *p* < 0.001, η^2^ = 0.017, (Fig. 1 & Suppl. Table 3). Stratifying by age of onset, both EOS patients, F(1,850) = 14.580, *p* < 0.001, η^2^ = 0.017, and AOS patients, F(1,1013) = 10.100, *p* = 0.002, η^2^ = 0.010, had significantly larger caudate than HC.

#### Adolescent patient/control analysis

**Fig. 2:**
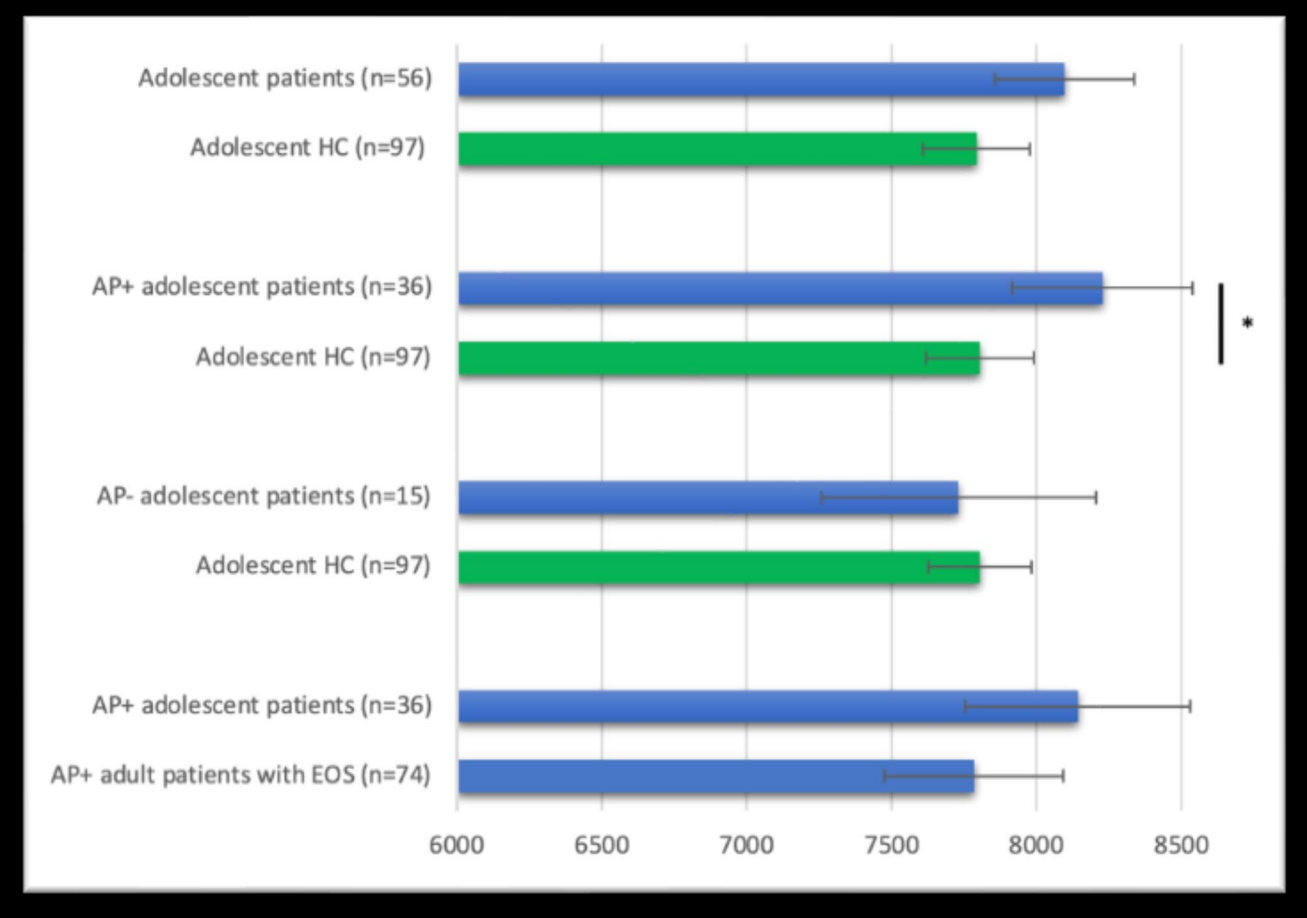
Caudate nucleus volume (in mm^3^) differences between adolescent patients with non-affective psychosis and healthy controls (HC) (up), between medicated (currently on antipsychotics; AP+) adolescent patients and HC as well as unmedicated (AP-) adolescent patients and HC (middle), and between AP + adolescent patients and AP + adult patients with early-onset schizophrenia (EOS) (down). Adjusted means from age-, sex, scanner- and estimated total intracranial volume-adjusted analyses of covariance are shown. Error bars represent the 95% confidence intervals calculated using standard errors. The green bars represent the HC group, while the blue bars represent the patient groups.

In the bivariate analysis of the adolescent samples, patients and HC did not differ in sex distribution, age or ICV (Table 3). In the sex-, age-, ICV-, and scanner-adjusted ANCOVA, patients had non-significantly larger caudate volume than HC, F(1,146) = 3.864, *p* = 0.051, η^2^ = 0.026 (Fig. 2 & Suppl. Table 4).

### Post-hoc analysis

#### Caudate specificity and laterality in EOS vs. AOS medicated adult patients

The results of the post-hoc analysis on all left and right subcortical structures are shown in Table 4. Both left (*p* < 0.001) and right (*p* = 0.003) caudate volumes were significantly larger in medicated patients with EOS relative to medicated patients with AOS, whereas none of the other analyzed subcortical structures differed between medicated patients with EOS and AOS.

#### Medicated and non-medicated adolescent patients vs. adolescent HC

Analyzing medicated patients only and HC, patients had significantly larger caudate volume than HC, F(1,126) = 5.220, *p* = 0.024, η^2^ = 0.040, whereas analyzing non-medicated patients only and HC, patients and HC did not differ in caudate volume, F(1,105) = 0.083, *p* = 0.774, η^2^ = 0.001 (Fig. 2 & Suppl. Tables 6 and 7).

#### Medicated adult patients with EOS vs. medicated adolescent patients

There was no significant difference in caudate volume between medicated adult patients with EOS and medicated adolescent patients, F(1,102) = 1.401, *p* = 0.239, η^2^ = 0.014 (Fig. 2 & suppl. material). When we excluded adolescent patients with psychosis not otherwise specified, there was still no difference in caudate volume between adolescent patients with EOS and adult patients with EOS, F(1,74) = 0.237, *p* = 0.628, η^2^ = 0.003.

## Discussion

In the present study, we showed that patients with SZ had larger caudate nucleus volumes than HC. This effect was greater in EOS compared to AOS. Further, current antipsychotic medication use as well as CPZ were associated with larger caudate volumes in EOS but not in AOS. Conversely, among patients on antipsychotics, caudate nucleus volumes were larger in EOS compared to AOS, whereas there was no such difference between unmedicated EOS and AOS. This may suggest that structural effects of antipsychotic medication interact with neurodevelopmental phase, which to our knowledge has not been previously reported. Interestingly, both right and left caudate were found to be larger in medicated EOS compared to AOS, whereas there were no volumetric differences for the other subcortical structures studied, which may suggest a caudate specificity. Finally, a direct comparison of caudate between medicated adults with EOS and medicated adolescent patients, with a mean duration of illness of two years, showed similar volumes. This may indicate that the suggested medication effect occurred already in adolescence close to the disease onset. This is further supported by our finding that, among adolescents, only medicated patients had larger caudate than HC.

Extensive research has been dedicated to studying the caudate nucleus in individuals with SZ, yet the findings have often presented conflicting results, revealing similar, smaller, or larger volumes when compared to HC. In a comprehensive meta-analysis involving adult patients with SZ, antipsychotic-naïve patients had significantly smaller caudate than HC, whereas medicated patients showed no difference compared to HC^13^. Further, in the large-scale multi-site ENIGMA (Enhancing NeuroImaging Genetics through Meta Analysis) study of adult SZ, patients did not significantly differ from HC in caudate volume^14^, whereas in the large-scale multi-site research by the Cognitive Genetics Collaborative Research Organization (COCORO), adult patients with SZ exhibited significantly larger caudate than HC^15^. Taken together, it seems that antipsychotic-naïve patients have smaller caudate than HC, whereas patients further along in the course of illness demonstrate similar or larger caudate compared to HC. This suggests that the enlargement in caudate volume may be related to medication usage or a progressive effect of the disorder. The current findings in our study reveal larger caudate volumes in AOS that are unrelated to medication use, as well as larger caudate volumes in EOS that can be attributed to medication. This may indicate that the larger caudate volumes in adult patients found in some previous studies are related to both the disorder itself and the use of antipsychotic medications. Discrepancies in previous research may be due to the inclusion of both medicated and unmedicated patients as well as the grouping of patients with AOS and EOS.

In the multi-site ENIGMA study of adolescent psychosis, the caudate was significantly larger in adolescent patients compared to HC, and that was the case even when patients with affective and non-affective psychosis were analyzed separately^12^. Of note, only patients currently on antipsychotics showed significantly larger caudate than HC, whereas currently unmedicated patients did not significantly differ relative to HC^12^, the latter possibly reflecting a preserved caudate volume at the disease onset. The similar caudate volumes in unmedicated adolescent patients and the smaller volumes in psychotic-naïve adult patients compare to their respective HC groups may be the disorder-related caudate volume at the disease onset and differentiates patients depending on the age of onset. A related question is whether the caudate volume is altered already before the onset of psychosis. The existing literature on this topic presents conflicting results with some studies showing unaltered^42,43^, unaltered but with abnormal shape^44^ or smaller^45^ volumes among high-risk individuals. In this context, Hannan et al. studied adolescents and young adults at ultra-high risk of developing psychosis, some of whom later converted to psychosis while others did not, and HC, and found no caudate volume difference between the three groups^43^.

In light of a reevaluation of the dopamine hypothesis regarding SZ, it has been proposed that a prominent aspect of the disorder involves an elevation in presynaptic dopamine synthesis within the striatum^7^. Notably, antipsychotic medications, which effectively block both dopamine D2 postsynaptic receptors and presynaptic autoreceptors, may trigger a compensatory response leading to an increase in dopamine synthesis^7^. This, in turn, may be shown as volume augmentation in volumetric studies. Moreover, it’s worth noting that alternative explanations for the observed increase in caudate volume associated with antipsychotic use have been discussed. These encompass factors such as microglial activation and heightened blood flow which may contribute to the observed structural changes^46^. Regardless of the specific mechanisms underpinning these findings, antipsychotic medication appear to exert a discernible impact on the caudate nucleus, particularly when administered during adolescence, a period characterized by robust neurodevelopment^21^.

Analyzing the age of onset of psychosis as a continuous variable, we identified a significant interaction between the age of onset and antipsychotic use on caudate volume. This finding supports the primary results of the present study, which demonstrated an EOS/AOS-by-antipsychotic use interaction on caudate volume. Additionally, although not statistically significant, we observed that among patients on antipsychotics, a younger age of onset of psychosis was associated with a larger caudate volume. This might suggest that the hypothesized effect of antipsychotics on caudate enlargement extends beyond adolescence, with a more pronounced enlargement associated with an earlier age of onset, even in adulthood. Further, the non-significant result from the MANCOVA, which accounts for the combined influence on all examined subcortical structures, indicates that the significant effect of age of onset is specific to the caudate nucleus. The effects on the other subcortical structures do not reach statistical significance and attenuate the overall effect when the seven structures are analyzed collectively, thereby leading to the non-significant outcome in the combined analysis.

The present study has certain limitations. First, we analyzed cross-sectional observational data, making it difficult to differentiate disease severity from treatment effect. Further, we had data on current medication use, but not cumulative antipsychotic medication use, or even more importantly for the current results, antipsychotic use during adolescence in adult patients with EOS. However, DOI was not associated with caudate volume, and medicated adult patients with EOS did not show larger caudate volume than adolescent patients which may be suggestive of a caudate enlargement already in adolescence. Further, to increase power in the analyses of our relatively small adolescent sample, we included patients with psychotic disorders not otherwise specified, whereas to increase specificity in our adult sample analyses, we only included patients with SZ. This may have influence the posthoc analysis where the adult AOS patients and the adolescent patients are directly compared. To address this concern, we reran the analysis including adolescent SZ patients only: the results did not change, still showing no caudate volume difference between the two patient groups. Next, although participants with histories of substance use (including alcohol use) disorders are not excluded from the TOP study, AUDIT and DUDIT scores did not significantly differ between EOS and AOS groups, nor were these scores correlated with caudate volumes (Table 1). However, EOS had a higher frequency of lifetime cannabis (but not of other substances) use than AOS; inserting the cannabis variable into the model, the results remained significant (Suppl. Material). These results reduce the likelihood that the observed associations between EOS/AOS and caudate volumes are confounded by substance use. In addition, even though both the adults and the adolescents were well-characterized, we cannot exclude the possibility that the observed associations are confounded by unknown factors. Finally, this is a volumetric study, and the biological explanation of the observed caudate nucleus enlargement remains necessarily speculative.

To conclude, adult patients with SZ showed significantly larger caudate nucleus volumes than HC, and most importantly, current antipsychotic medication use was associated with significantly larger caudate in EOS but not in AOS. These findings were consistent for both the left and right caudate volumes. The results may suggest a dual influence on caudate volume, with implications both related to the pathology of schizophrenia itself and the potential impact of medication, the latter restricted to patients with EOS. Based on the lack of volumetric differences between adult patients with EOS and adolescent patients, we further suggest that this increase might occur already in adolescence in proximity to the onset of psychosis. These novel findings shed light on the concept that the degree of structural plasticity exhibited by the caudate during antipsychotic treatment may be contingent upon the developmental stage of the patients. Further research is needed to elucidate the underlying pathophysiological mechanisms and clinical significance of caudate enlargement in the context of schizophrenia.

## Electronic supplementary material

Below is the link to the electronic supplementary material.

Supplementary Material 1

## Data Availability

Data supporting the findings of the present study have repository at NORMENT/Oslo University Hospital. Restrictions apply to the availability of data and are thereby not publicly available. Data can be made available under reasonable request to the corresponding author and with permission of NORMENT/Oslo University Hospital, in accordance with the ethics agreements/research participants consent.
