# Supplementary material for: Caudate nucleus volume in medicated and unmedicated patients with early- and adult-onset schizophrenia
Source: Sci Rep. 2024 Oct 1;14:22755. doi: 10.1038/s41598-024-73322-x (PMC11445249; doi:10.1038/s41598-024-73322-x)
Supplement: Supplementary file 1 — Supplementary Material 1 [file 41598_2024_73322_MOESM1_ESM.docx]

**Suppl. material**

**Caudate nucleus volume in medicated and unmedicated patients with early- and adult-onset schizophrenia**

Dimitrios Andreou, Kjetil Nordbø Jørgensen, Stener Nerland, Tereza Calkova, Lynn Mørch-Johnsen, Runar Elle Smelror, Laura A. Wortinger, Mathias Lundberg, Hannes Bohman, Anne Margrethe Myhre, Erik G. Jönsson, Ole A. Andreassen, Ingrid Agartz

**Age of onset and age distributions**


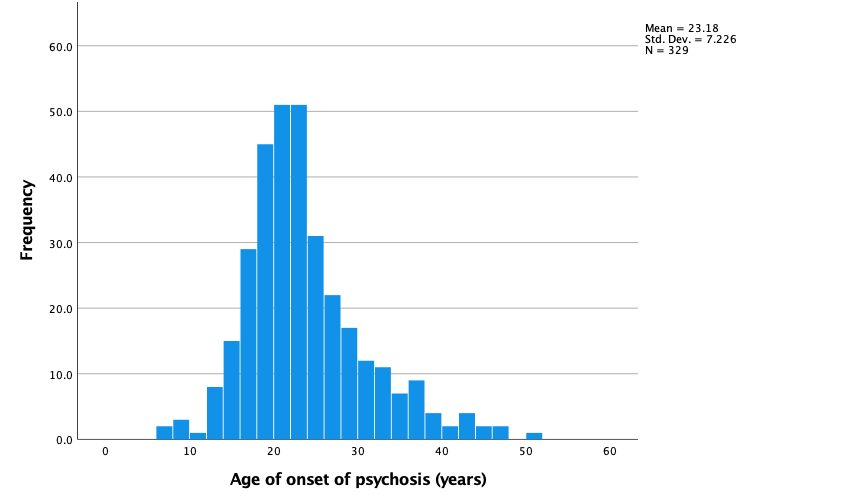

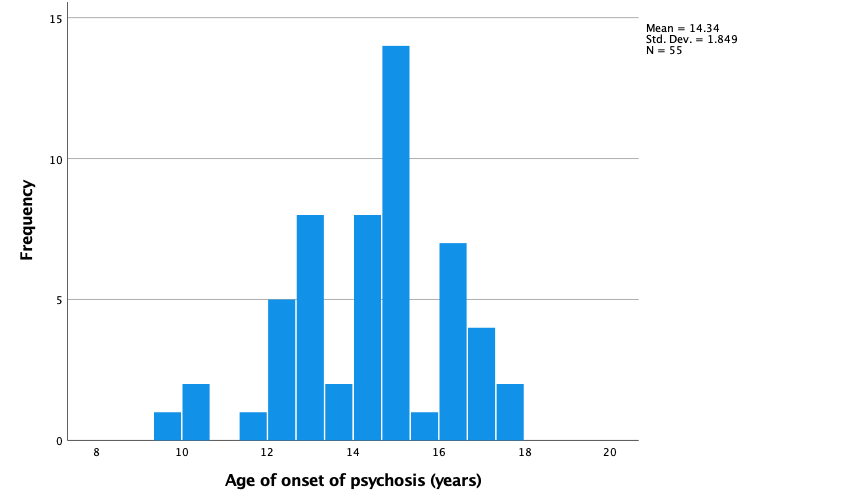


**Suppl. Figure 1.** Distribution of age of onset of psychosis in adult patients with schizophrenia spectrum disorders (left) and adolescent patients with non-affective psychosis (right)


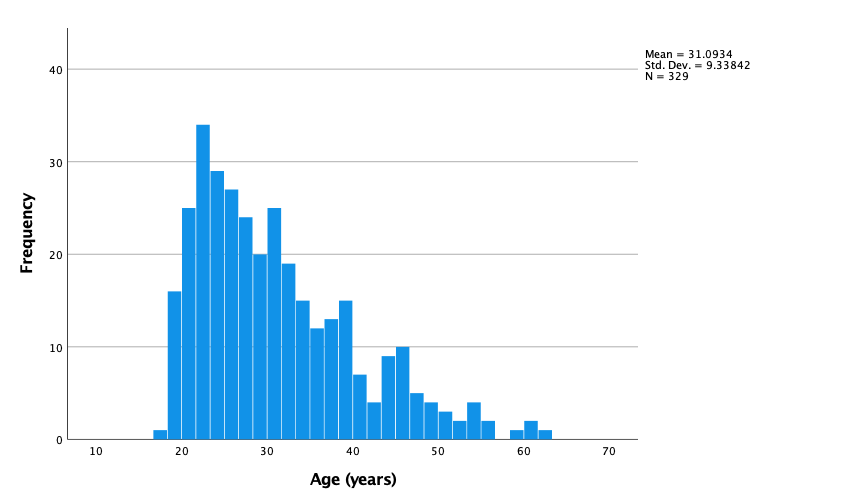

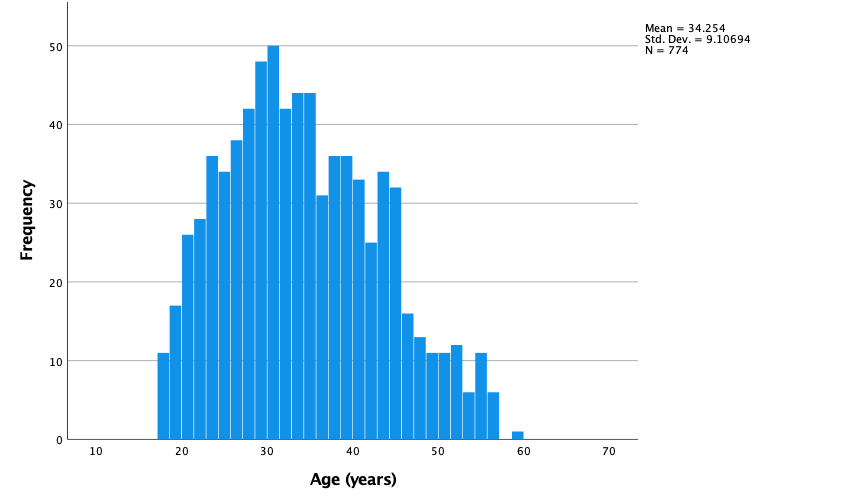


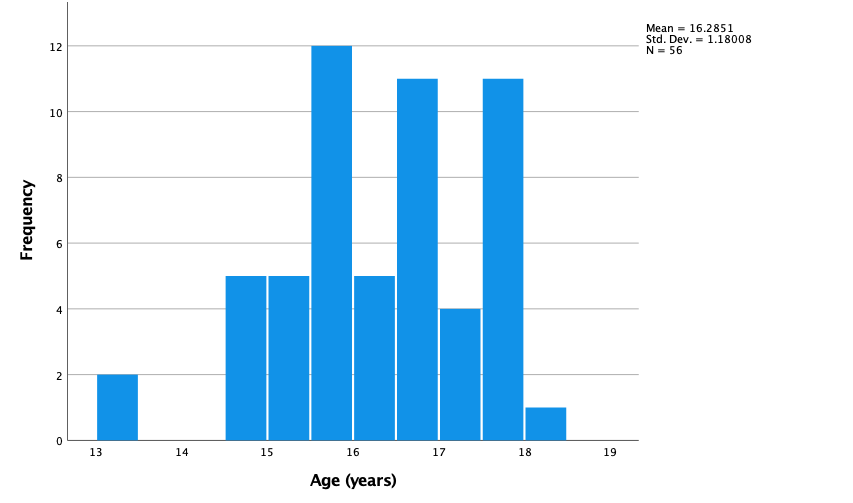

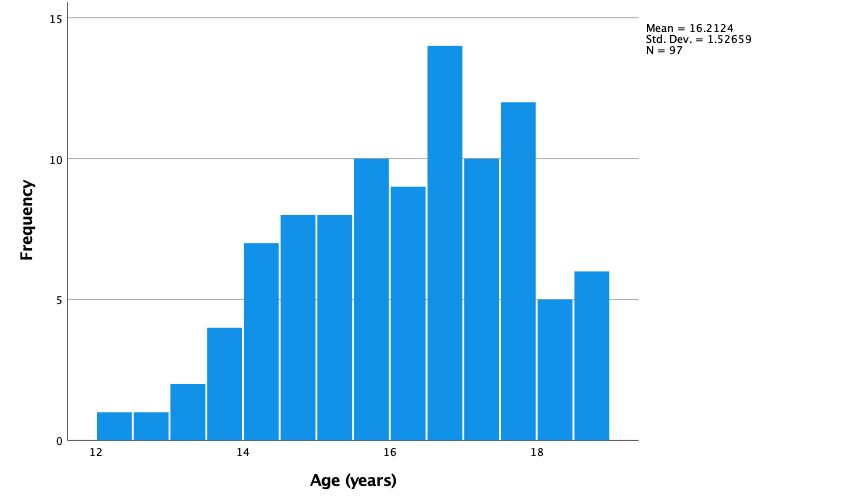


**Suppl. Figure 2.** Age distribution in adult patients with schizophrenia spectrum disorders (up-left), adult HC (up-right), adolescent patients with non-affective psychosis (down-left) and adolescent HC (down-right)

**Main analysis**

***Adult patient analysis (model 1)***

There was homogeneity of variances evaluated with Levene’s test, p=0.389. The residuals of the overall model were approximately normally distributed determined by visual inspection. There was one outlier with studentized residual greater than three standard deviations. This case did not exhibit high leverage or high Cook's distance value and was included in the final model. In a model where this case was excluded, there was still as a significant EOP/AOP effect (p=0.008).

| **ANCOVA (n=329)** | **F** | **P value** | **Partial eta^2^** |
| --- | --- | --- | --- |
| EOS/AOS | 7.200 | 0.008 | 0.022 |
| Age | 0.042 | 0.839 | <0.001 |
| Sex | 0.874 | 0.350 | 0.003 |
| Scanner | 10.600 | <0.001 | 0.062 |
| ICV | 104.470 | <0.001 | 0.245 |

**Suppl. Table 1**. The results of the analysis of covariance (ANCOVA) on caudate among patients with schizophrenia (model 1)

EOS: early-onset schizophrenia, AOS: adult-onset schizophrenia, ICV: estimated total intracranial volume

***Adult patient analysis (model 2)***

There was homogeneity of variances evaluated with Levene’s test, p=0.595. The residuals of the overall model were approximately normally distributed determined by visual inspection. There were two outliers with studentized residuals greater than three standard deviations. These cases did not exhibit high leverage or high Cook's distance value and were included in the final model. In a model where these cases were excluded, there was still as a significant EOP/AOP-by-antipsychotic medication use interaction (p<0.001).

***Adult patient analysis (model 3)***

There was homogeneity of variances evaluated with Levene’s test, p=0.444 The residuals of the overall model were approximately normally distributed determined by visual inspection. There were no outliers with studentized residuals greater than three standard deviations.

| **ANCOVA (n=232)** | **F** | **P value** | **Partial eta^2^** |
| --- | --- | --- | --- |
| EOS/AOS | 0.571 | 0.451 | 0.003 |
| Use of antipsychotics | 10.917 | 0.001 | 0.047 |
| EOS/AOS-by-use of antipsychotics | 7.512 | 0.008 | 0.031 |
| Age | 3.939 | 0.048 | 0.018 |
| Sex | 0.050 | 0.824 | <0.001 |
| Scanner | 8.953 | <0.001 | 0.075 |
| ICV | 107.954 | <0.001 | 0.328 |
| DOI | 1.147 | 0.285 | 0.005 |
| DUP | 0.459 | 0.499 | 0.002 |

**Suppl. Table 2**. The results of the analysis of covariance (ANCOVA) on caudate among patients with schizophrenia (model 3)

EOS: early-onset schizophrenia, AOS: adult-onset schizophrenia, ICV: estimated total intracranial volume, DOI: duration of illness, DUP: duration of untreated psychosis

***Adult patient/control analysis***

There was homogeneity of variances evaluated with Levene’s test, p=0.223. The residuals of the overall model were approximately normally distributed determined by visual inspection. There were eight outliers with studentized residuals greater than three standard deviations. These cases did not exhibit high leverage or high Cook's distance value and were included in the final model. In a model where these cases were excluded, there was still as a significant main effect of patient/control status on (p<0.001).

| **ANCOVA (n=1103)** | **F** | **P value** | **Partial eta^2^** |
| --- | --- | --- | --- |
| Patient/control | 19.372 | <0.001 | 0.017 |
| Age | 45.316 | <0.001 | 0.040 |
| Sex | 2.146 | 0.143 | 0.002 |
| Scanner | 26.086 | <0.001 | 0.045 |
| ICV | 367.903 | <0.001 | 0.251 |

**Suppl. Table 3**. The results of the analysis of covariance (ANCOVA) on caudate among adult patients and healthy controls

ICV: estimated total intracranial volume

***Adolescent patient/control analysis***

There was homogeneity of variances evaluated with Levene’s test, p=0.352. The residuals of the overall model were approximately normally distributed determined by visual inspection. There were no outliers with studentized residuals greater than three standard deviations.

| **ANCOVA (n=153)** | **F** | **P value** | **Partial eta^2^** |
| --- | --- | --- | --- |
| Patient/control | 3.864 | 0.051 | 0.026 |
| Age | 0.760 | 0.385 | 0.005 |
| Sex | 2.278 | 0.133 | 0.015 |
| Scanner | 7.492 | <0.001 | 0.093 |
| ICV | 23.813 | <0.001 | 0.140 |

**Suppl. Table 4**. The results of the analysis of covariance (ANCOVA) on caudate among adolescent patients and healthy controls

ICV: estimated total intracranial volume

***Analyses on left and right caudate volumes***

As shown in Table 1, the use of antipsychotics was positively correlated with the caudate volume in EOS, r_pb_=0.322, p=0.003, but not in AOS, r_pb_=0.037, p=0.564. The use of antipsychotics was positively correlated with both the left and the right caudate volumes in EOS (r_pb_=0.360, p<0.001 and r_pb_=0.272, p=0.013, respectively), but not in AOS (r_pb_=0.027, p=0.671 and r_pb_=0.045, p=0.483, respectively).

As shown in Table 1, CPZ were positively correlated with the caudate volume in EOS, r_s_=0.248, p=0.033, but not in AOS, r_s_=-0.045, p=0.507. CPZ were positively correlated with both the left and the right caudate volumes in EOS (r_s_=0.234, p=0.045 and r_s_=0.251, p=0.031, respectively), but not in AOS (r_s_=-0.041, p=0.548 and r_s_=-0.036, p=0.603, respectively).

Applying ANCOVAs adjusted for sex, age, scanner and ICV, we first explored the main effect of diagnostic status (EOS/AOS) on left and right caudate volumes (model 1). As for the whole caudate (left + right, p=0.008), there were similarly significant main effects of EOS/AOS on both left (F(1,322)=7.353, p=0.007, η^2^=0.022) and right (F(1,322)=6.179, p=0.013, η^2^=0.019) caudate volumes.

We then explored the main and interaction effects of EOS/AOS and antipsychotic medication use on left and right caudate (model 2; main model). There was a significant EOS/AOS-by-antipsychotic medication interaction (p=0.001) on left caudate: among patients with EOS, there was a statistically significant effect of antipsychotic medication use on left caudate, F(1,320)=15.436, p<0.001, η^2^=0.046, whereas among patients with AOS, there was no such effect, F(1,320)=0.077, p=0.782, η^2^ <0.001. Similarly, there was a significant EOS/AOS-by-antipsychotic medication interaction (p=0.02) on right caudate: among patients with EOS, there was a statistically significant effect of antipsychotic medication use on right caudate, F(1,320)=9.351, p=0.002, η^2^=0.028, whereas among patients with AOS, there was no such effect, F(1,320)=0.202, p=0.348, η^2^ =0.001.

Further, we investigated the putative differences in left and right caudate volumes between patients and HC. In sex-, age-, ICV-, and scanner-adjusted ANCOVAs, as for the whole caudate volume (p<0.001), patients had significantly larger left, F(1,1096)=16.168, p<0.001, η^2^=0.015, and right, F(1,1096)=20.489, p<0.001, η^2^=0.018, caudate volumes than HC. As for the whole caudate volume, stratifying by age of onset, both EOS patients, F(1,850)=13.335, p<0.001, η^2^=0.015, and AOS patients, F(1,1013)=7.690, p=0.006, η^2^=0.008, had significantly larger left caudate than HC. The corresponding statistics for right caudate were F(1,850)=14.190, p<0.001, η^2^=0.016 for EOS and F(1,1013)=11.550, p<0.001, η^2^=0.011 for AOS.

***PANSS subscale scores***

As for the total PANSS score (Table 1), PANSS general, negative and positive symptom scores were not significantly correlated with the caudate volume in EOS (p=0.128, p=0.404 and p=0.529, respectively) or AOS (p=0.983, p=0.132 and p=0.875, respectively). Analysis of the left and right caudate volumes similarly revealed no significant correlations with the PANSS subscale scores (data not shown).

***Lifetime substance use analysis in EOS and AOS***

|  | **EOS** | | | **AOS** | | | **P-value^1^** |
| --- | --- | --- | --- | --- | --- | --- | --- |
|  | **Never** | **<10 times** | **>10 times** | **Never** | **<10 times** | **>10 times** |  |
| **Hallucinogens** | 71.2 | 16.9 | 11.9 | 73 | 20.6 | 6.3 | 0.333 |
| **Cocaine** | 65 | 25 | 10 | 68.8 | 22.2 | 9 | 0.861 |
| **Opiates** | 84.7 | 13.6 | 1.7 | 90.5 | 5.8 | 3.7 | 0.136 |
| **Stimulants** | 65.6 | 11.5 | 23 | 67 | 18.6 | 14.4 | 0.176 |
| **Cannabis** | 18 | 29.5 | 52.5 | 36 | 20.6 | 43.4 | **0.028** |
| **Sedatives** | 81 | 12.1 | 6.9 | 85.1 | 5.9 | 9 | 0.308 |

**Suppl. Table 5.** Lifetime use of substances in patients with EOS and AOS

^1^Chi-square test or Fisher’s exact test

Patients with EOS showed a significantly higher frequency of lifetime cannabis use than AOS, with no such differences when we studied other substances (Suppl. Table 5). We reran the ANCOVA presented in the main text (model 3) inserting the cannabis use variable. There was still a significant EOS/AOS-by-antipsychotic medication interaction (p=0.012). Among patients with EOS, there was a statistically significant effect of antipsychotic medication use on caudate, F(1,239)=8.057, p=0.005, whereas among patients with AOS, there was no such effect, F(1,239)<0.001, p=0.996.

***Medication-caudate volume correlations***

As presented in Table 1, CPZ exhibit a positive correlation with caudate volume in the EOS group, but not in the AOS group. These correlations are depicted in Supplementary Figure 3. Furthermore, the use of antipsychotics, represented as a binary variable, also shows a significant correlation with caudate volume in the EOS group, but not in the AOS group. However, due to the nature of point-biserial correlations, this relationship cannot be visualized in the same manner.

**
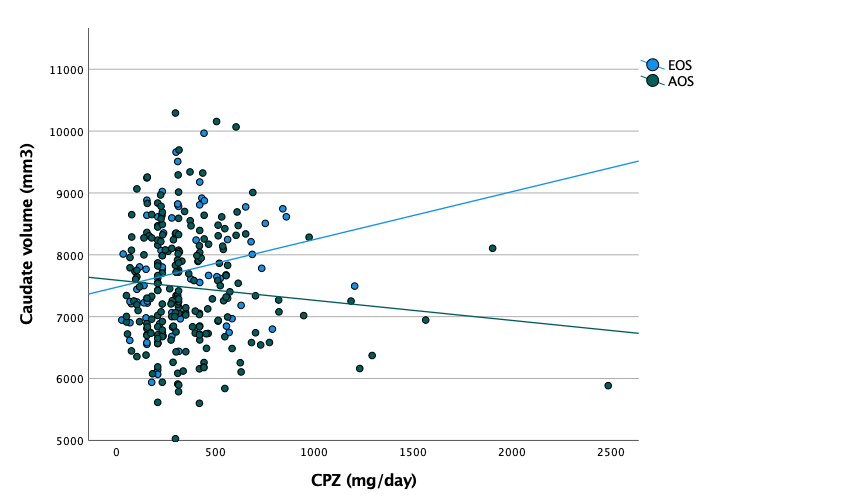
**

**Suppl. Figure 3.** Scatterplot of caudate volumes and CPZ in EOS and AOS. Fit method: Linear

**Posthoc analysis**

***Medicated adolescent patient/control analysis***

There was homogeneity of variances evaluated with Levene’s test, p=0.405. The residuals of the overall model were approximately normally distributed determined by visual inspection. There were no outliers with studentized residuals greater than three standard deviations.

| **ANCOVA (n=133)** | **F** | **P value** | **Partial eta^2^** |
| --- | --- | --- | --- |
| Patient/control | 5.220 | 0.024 | 0.040 |
| Age | 1.933 | 0.167 | 0.015 |
| Sex | 0.470 | 0.494 | 0.004 |
| Scanner | 5.841 | 0.004 | 0.085 |
| ICV | 21.639 | <0.001 | 0.147 |

**Suppl. Table 6**. The results of the analysis of covariance (ANCOVA) on caudate among medicated adolescent patients and healthy controls

ICV: estimated total intracranial volume

***Non-medicated adolescent patient/control analysis***

There was homogeneity of variances evaluated with Levene’s test, p=0.419. The residuals of the overall model were approximately normally distributed determined by visual inspection. There were no outliers with studentized residuals greater than three standard deviations.

| **ANCOVA (n=113)** | **F** | **P value** | **Partial eta^2^** |
| --- | --- | --- | --- |
| Patient/control | 0.083 | 0.774 | 0.001 |
| Age | 0.168 | 0.683 | 0.002 |
| Sex | 0.299 | 0.586 | 0.003 |
| Scanner | 7.901 | <0.001 | 0.131 |
| ICV | 15.064 | <0.001 | 0.125 |

**Suppl. Table 7**. The results of the analysis of covariance (ANCOVA) on caudate among non-medicated adolescent patients and healthy controls

ICV: estimated total intracranial volume

***Medicated adolescent patients vs. medicated adult patients with EOS analysis***

The residuals of the overall model were approximately normally distributed determined by visual inspection. There were no outliers with studentized residuals greater than three standard deviations. There was a deviation from the homogeneity of variances assumption assessed with Levene’s test (p=0.023) indicating unequal variances. We computed bootstrapped bias-corrected and accelerated (BCa) 95% confidence intervals (CI). As in the model reported in the main text, there was still no statistically significant difference in caudate volumes between the two patient groups, BCa 95% CI, -959.415 to 245.105, p=0.234.

|  | **MAGNETOM Sonata** | **Signa HDxt** | **Discovery MR750 (Oslo)** | **Discovery MR750 (Stockholm)** |
| --- | --- | --- | --- | --- |
| **Sample** | TOP | TOP/YTOP | TOP/YTOP | SCAPS |
| **Field strength** | 1.5T | 3T | 3T | 3T |
| **Adults (N; patients/controls)** | 496  (205/291) | 351  (73/278) | 256  (51/205) | - |
| **Adolescents  (N; patients/controls)** | - | 55  (19/36) | 52  (18/34) | 46  (19/27) |
| **Sequence name** | MPRAGE | FSPGR | BRAVO | BRAVO |
| **Echo time (ms)** | 3,93 | MinFull | 3,18 | 3,06 |
| **Repetition time (ms)** | 2730 | 7,8 | 8,16 | 7,98 |
| **Inversion time (ms)** | 1000 | 450 | 450 | 450 |
| **Flip angle** | 7 ° | 12 ° | 12 ° | 12 ° |
| **Voxel size (mm3)** | 1.33x0.94x1 | 1x1x1.2 | 1x1x1 | 1x1x1.2 |

**Suppl. Table 8.** An overview of the scanner systems used to acquire each sample and their T1-weighted MRI sequences

To illustrate the segmentation quality for the caudate, we selected three random participants from each dataset (a total of nine participants) and visualized their caudate segmentations as a blue outline with opacity of 0.5 overlaid onto their T1-weighted (T1w) images (orig.mgz). Note that for the 1.5T scanner, orig.mgz is a robust average of the two acquired T1w images. In these plots, each column represents a unique participant and the rows show the coronal, axial, and sagittal views of the segmentations. The segmentations closely followed the contrast between the caudate and surrounding tissue (white matter and cerebrospinal fluid) despite large interindividual variation in the extension of the ventricles, as well as the size, shape, and relative placement of the caudate (Suppl. Figure 4).

**
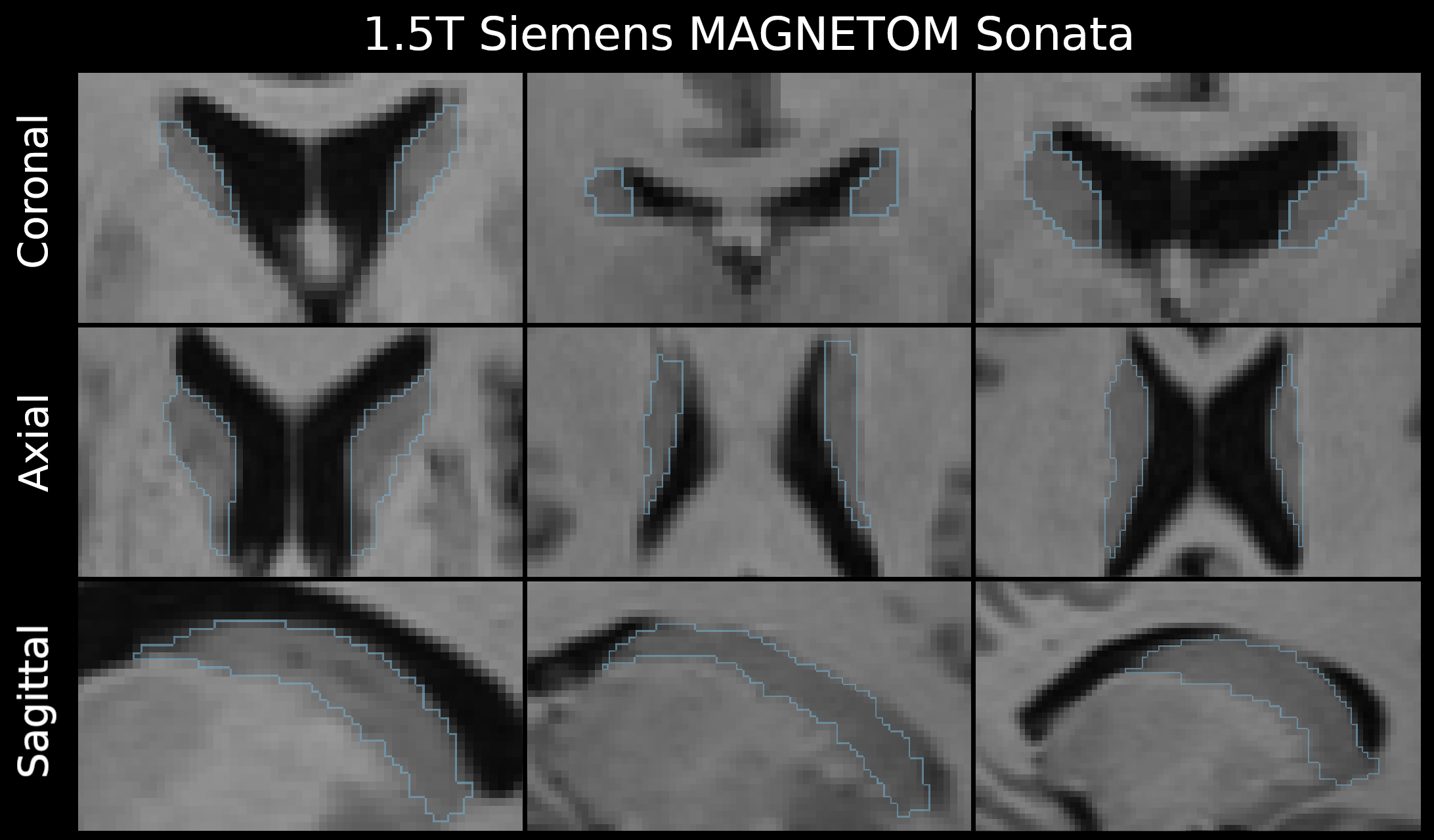
**

**
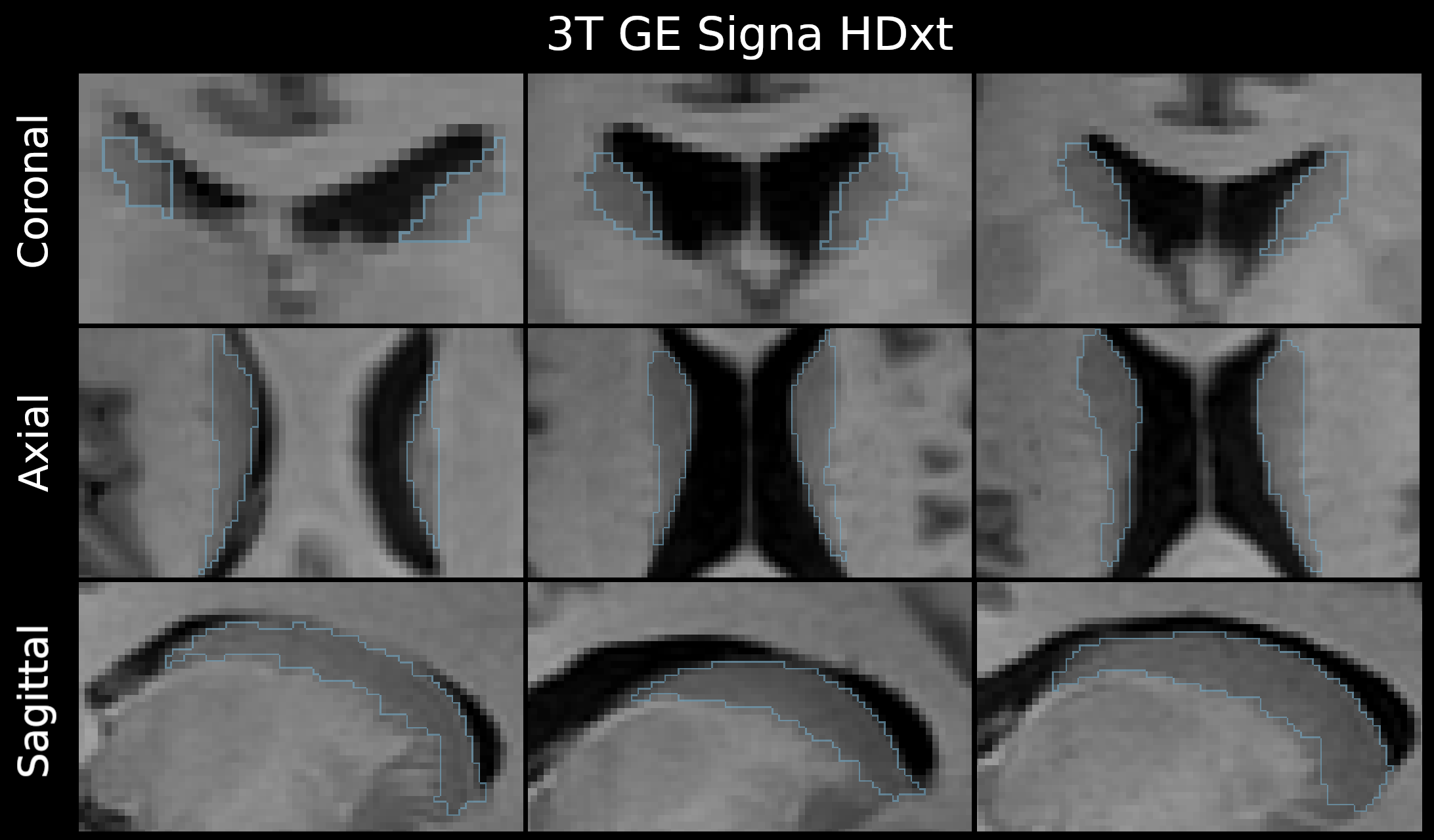
**

**
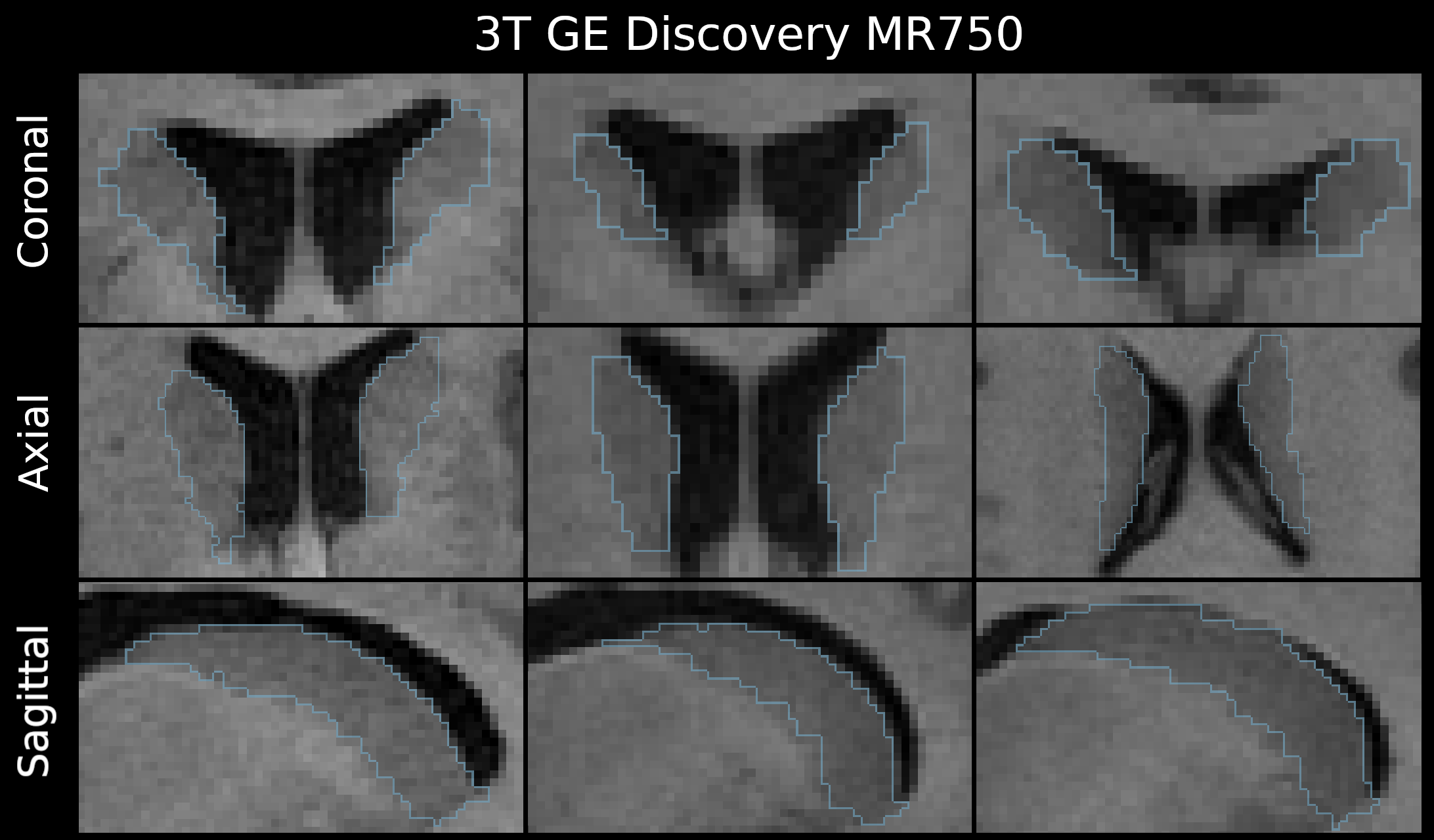
**

**Suppl. Figure 4.** Visualization of caudate segmentations as a blue outline with opacity of 0.5 overlaid onto the T1-weighted images
